# Correction to RING‐Type E3 Ligase OsRFPH2‐6 Ubiquitinates Nuclear Factor Y Subunit OsNF‐YA1 to Suppress Rice Immunity

**DOI:** 10.1111/mpp.70216

**Published:** 2026-02-11

Y. Bi, L. Tariq, H. Wang, Y. Yan, D. Li, and F. Song. 2025. “RING‐type E3 Ligase OsRFPH2‐6 Ubiquitinates Nuclear Factor Y Subunit OsNF‐YA1 to Suppress Rice Immunity.” *Molecular Plant Pathology* 26, no. 12: e70193. https://doi.org/10.1111/mpp.70193.

Upon publication of the above paper, the authors identified an inadvertent error in Figure 8. Due to poor quality of the original Figure 8, a new version with higher quality was requested during proofreading. Unfortunately, an incorrect Figure 8 was uploaded as an attachment to the Wiley Proofreading System, which led to this error.

The correct version of Figure 8 is shown below. This correction does not alter any results and conclusions of the study reported in the paper.

**FIGURE 8:**
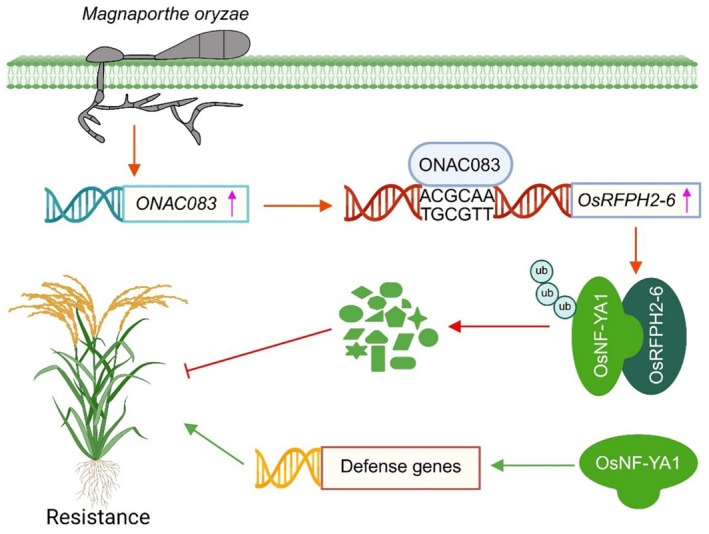
Proposed model for the ONAC083‐OsRFPH2‐6‐OsNF‐YA1 regulatory network in rice immunity. OsRFPH2‐6, an E3 ligase that negatively regulates rice immunity against *Magnaporthe oryzae*, is transcriptionally activated by ONAC083, a NAC transcription factor that also functions as a negative regulator of rice immunity to *M. oryzae*. Upon *M. oryzae* infection, *ONAC083* expression is upregulated and subsequently activates *OsRFPH2‐6* transcription by binding to the ACGCAA *cis*‐element in its promoter region. OsRFPH2‐6 then interacts with OsNF‐YA1, mediating its ubiquitination and subsequent degradation through the UPS pathway. This molecular cascade ultimately disrupts the positive regulatory role of OsNF‐YA1 in rice resistance to *M. oryzae*, thereby contributing to the negative regulation of rice immunity.

We apologise for this error.

